# Spatial and Temporal Impacts of Socioeconomic and Environmental Factors on Healthcare Resources: A County-Level Bayesian Local Spatiotemporal Regression Modeling Study of Hospital Beds in Southwest China

**DOI:** 10.3390/ijerph17165890

**Published:** 2020-08-13

**Authors:** Chao Song, Yaode Wang, Xiu Yang, Yili Yang, Zhangying Tang, Xiuli Wang, Jay Pan

**Affiliations:** 1 State Key Laboratory of Oil and Gas Reservoir Geology and Exploitation, School of Geoscience and Technology, Southwest Petroleum University, Chengdu 610500, China; available1516@163.com (Y.W.); tzycd@163.com (Z.T.); 2 West China School of Public Health (West China Fourth Hospital), Sichuan University, Chengdu 610041, China; wang_xiuli@scu.edu.cn; 3 State Key Laboratory of Resources and Environmental Information System (LREIS), Institute of Geographic Sciences and Natural Resources Research, Chinese Academy of Sciences, Beijing 100101, China; 4 China Science and Technology Exchange Center, Division of Policy Study, Beijing 100045, China; yangxiu731@126.com; 5 West China Research Center for Rural Health Development, Sichuan University, Chengdu 610041, China; yyl115@hotmail.com

**Keywords:** Bayesian STVC model, healthcare resources, geographical inequality, hospital beds, socioeconomic and environmental factors, spatiotemporal nonstationarity, health planning, China

## Abstract

Comprehensive investigation on understanding geographical inequalities of healthcare resources and their influencing factors in China remains scarce. This study aimed to explore both spatial and temporal heterogeneous impacts of various socioeconomic and environmental factors on healthcare resource inequalities at a fine-scale administrative county level. We collected data on county-level hospital beds per ten thousand people to represent healthcare resources, as well as data on 32 candidate socioeconomic and environmental covariates in southwest China from 2002 to 2011. We innovatively employed a cutting-edge local spatiotemporal regression, namely, a Bayesian spatiotemporally varying coefficients (STVC) model, to simultaneously detect spatial and temporal autocorrelated nonstationarity in healthcare-covariate relationships via estimating posterior space-coefficients (SC) within each county, as well as time-coefficients (TC) over ten years. Our findings reported that in addition to socioeconomic factors, environmental factors also had significant impacts on healthcare resources inequalities at both global and local space–time scales. Globally, the personal economy was identified as the most significant explanatory factor. However, the temporal impacts of personal economy demonstrated a gradual decline, while the impacts of the regional economy and government investment showed a constant growth from 2002 to 2011. Spatially, geographical clustered regions for both hospital bed distributions and various hospital bed-covariates relationships were detected. Finally, the first spatiotemporal series of complete county-level hospital bed inequality maps in southwest China was produced. This work is expected to provide evidence-based implications for future policy making procedures to improve healthcare equalities from a spatiotemporal perspective. The employed Bayesian STVC model provides frontier insights into investigating spatiotemporal heterogeneous variables relationships embedded in broader areas such as public health, environment, and earth sciences.

## 1. Introduction

Rational allocation of healthcare resources has been addressed as an essential goal to be achieved throughout healthcare system reforms in a worldwide range [1,2], for which evaluating the inequalities of healthcare resources remains a prevalent issue, especially from the perspectives of spatial dimension [3,4,5] and temporal dimension [6,7], as well as from both dimensions [8]. The number of hospital beds has been commonly addressed as a proxy of healthcare resources [9]. In China, the geographical inequalities of hospital bed distribution remain a noteworthy problem and are found to be aggravated at finer-scaled geospatial levels [7]. Aware of this persistent issue, most studies on China’s hospital bed inequalities in the literature, however, have been conducted merely at national [10] or provincial [11,12] levels instead of at a finer-scaled county level [13]. The least equity in healthcare resources was reported in western China [14]. According to the latest report, 823,200 hospital beds in western China in 2009 only accounted for 26.06% of the total nationwide hospital beds, while demonstrating a massive gap in this aspect compared with all the other regions in China (e.g., eastern region 43.35%, central region 30.59%) [15]. Several studies conducted in southwestern China have reported notable disparities in healthcare resources at the provincial level, with a couple of provinces presenting inadequate resources below the nationwide average [12,13,16,17]. In spite of these findings, no studies conducted in southwestern China have investigated the inequality in hospital beds at a finer-scaled administrative level, namely the county level, not to mention the conduction of such investigations from an integrated spatial-temporal perspective.

Apart from the investigation on unevenly distributed healthcare resources, the exploration of potential factors influencing such inequality issues also cannot be ignored as a meaningful strategy for optimizing healthcare resource allocation from a holistic perspective. Previous studies have identified multiple socioeconomic factors affecting the distribution of hospital beds among regions [17,18]. Specifically, Qin et al. found that GDP per capita, population size, and the level of public spending all played essential roles in accelerating the diffusion of healthcare resources in respect of hospital beds across various regions in China [19]. On the other hand, Guo et al. found that GDP, income, education, financial expenditure, and population size had different impacts on the concentration of hospital beds in different regions of China [20]. While Pan et al. identified residents’ saving deposits and government revenue as the most influential socioeconomic indicators [13], Ceccherini-Nelli et al. found another long-term cyclical relationship between hospital beds and a list of socioeconomic factors such as real GDP, base discount rates, and the rate of unemployment in a number of regions [21]. In an attempt to investigate the relationship between population growth and hospital beds expansion, Yu et al. reported that the growth rate of hospital beds presented slower in regions with rapid population growth, while becoming faster in regions with comparatively slower population growth rates [14]. Another group of researchers also explored the spatial impacts of socioeconomic factors on hospital beds taking a list of socioeconomic factors into consideration including the proportion of the elderly population, proportion of the urban population, GDP per capita, and health expenditure [17]. It is noteworthy that all these previous studies failed to conduct comprehensive investigations into hospital beds from a socioeconomic perspective with only very limited socioeconomic factors to be taken into consideration. In an attempt to bridge such research gaps embedded in previous literature, a complete space–time dataset which contains various county-level socioeconomic factors in China was recently developed and proposed by Song et al. in a newly published study which is believed to serve as a potent tool for exploring the impacts of various socioeconomic factors on hospital beds in a much more comprehensive manner [22].

In addition to socioeconomic status, researchers are also aware of the critical role that environmental condition has been playing in affecting hospital bed distributions. For instance, Song et al. conducted a study on the spillover effect of hospital beds in China by involving two environmental factors for investigation, namely the proportion of mountainous areas and traffic environment [8]. In another study, Ye et al. explored the impact of highway mileage as a traffic-related factor on hospital beds across different regions in China [17], based on which Wu et al., further identified topographic relief to be the most influential environmental factor on hospital bed distributions in Sichuan province of China [16], which exacerbated the long-existing imbalanced allocation of hospital beds in those regions, as well as tended to become a limiting factor over time. Based on the previous literature, we are aware that no comprehensive study in China that has ever investigated the spatiotemporal effects of both socioeconomic and environmental factors on hospital bed inequalities via adopting county as the lowest administrative level.

We are also aware of a constantly proposed assumption embedded in previous studies that the healthcare-covariates relationship remains homogeneous over the entire study area and time frame, which is known as stationarity in the field of statistics as a global-scaled assumption. For example, the commonly used Gini coefficient and Theil index for measuring the uneven distribution of hospital beds should only be regarded as a global-scaled coefficient in terms of evaluating the equality of healthcare resource allocation [14,23,24,25]. Likewise, conventional regression methods are only able to estimate the overall correlated effect of each covariate on hospital bed outcomes based on such a global-scaled assumption [13]. It should be noted that a list of problems is likely to be induced as the result of such a global-scaled assumption. The most important one is, in the real world, that the heterogeneity of both spatial and temporal scales should be taken into consideration when evaluating such healthcare-covariate relationships, especially for large-scale geospatial studies. In the field of spatial statistics, such local-scaled spatiotemporal heterogeneous variable relationships are called spatiotemporal nonstationarity [26,27]. Based on previous literature, the investigation into hospital bed-covariate nonstationary relationships has only been conducted in a single spatial dimension [11,16,17]. Therefore, a well-rounded investigation of the local-scaled spatiotemporal heterogeneous associations among county-level hospital beds is urgently needed via the incorporation of both socioeconomic and environmental factors within the study area of southwest China.

Based on fully understanding the issues as discussed above, we proposed two hypothetical theories related to healthcare resources in the study area: (1) Apart from socioeconomic factors, the county-level inequalities of healthcare resources were also associated with environmental factors. (2) The impacts of explanatory variables (e.g., socioeconomic and environmental factors) on healthcare resources outcomes were not homogeneous, but were instead heterogeneous at both space (e.g., county) and time (e.g., year) scales. Striving to verify these two hypothetical theories, in this study, we employed a state-of-the-art local spatiotemporal regression approach, namely, a Bayesian spatiotemporally varying coefficients (STVC) model, to innovatively quantify and characterize the spatial and temporal heterogeneous associations of county-level hospital beds with multiple socioeconomic and environmental covariates across southwest China from 2002 to 2011. The STVC model is a Bayesian-based local spatiotemporal regression proposed for space–time big data, which was aimed at simultaneous detection of spatial and temporal autocorrelated nonstationarity in heterogeneous response-covariate variables relationships [28,29]. Both Bayesian statistics theories and hierarchical modeling frameworks are adopted in this real “full map” modeling approach, along with a space–time independent nonstationary assumption that is achieved by separately estimating posterior local-scale coefficients over different space areas and time points [29].

Three objectives were included in this study. Firstly, to identify the essential covariates affecting hospital beds considering both socioeconomic and environmental aspects on a global scale. Secondly, to deeply explore the local-scaled spatial and temporal random effects for both hospital bed- covariates relationships and hospital bed distribution alone. Thirdly, to innovatively develop a series of complete and spatiotemporal maps of hospital bed inequality in southwest China at the county level.

## 2. Materials and Methods

### 2.1. Study Area and Data

**Figure 1:**
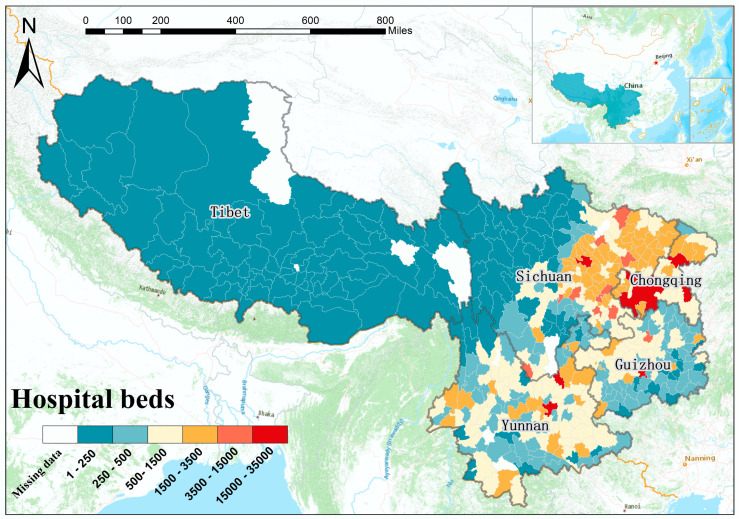
Geographical distribution of the original county-level hospital beds in the study area of southwest China in 2002.

The study area of southwest China is located between 97°21′–110°11′ east longitude and 21°08′–33°41′ north latitude, which is known as one of the seven geographical divisions accredited to China. The land area of southwest China is 2.5 million square kilometers, accounting for 24.5% of the whole of China. The complex terrain of southwest China mainly consists of plateau and mountainous areas. Southwest China covers five administrative regions, namely Sichuan province, Guizhou province, Yunnan province, Tibet province, and Chongqing municipality. Figure 1 illustrates the original geographical distribution of county-level hospital beds across southwest China in 2002. Considering the population impact, the number of hospital beds per ten thousand people was employed as the target variable of interest.

Correspondingly, an administrative county-level space–time dataset was innovatively developed in the first step via the integration of a complete list of socioeconomic and environmental factors that might potentially influence healthcare resources in southwest China over ten years. To be specific, we collected a total number of 32 county-level variables, including 20 socioeconomic factors and 12 environmental factors, as a list of potential covariates influencing healthcare resource allocations in southwest China, which were summarized in Table 1. Among them, the hospital beds and socioeconomic data were retrieved from China’s first official published county-level socioeconomic statistics dataset, which had been originally collected from the China County Statistical Yearbook, the China Statistical Yearbook for Regional Economy, and the China City Statistical Yearbook [22]. However, as China’s county-level statistical yearbooks have been upgraded by removing a variety of critical variables after 2003, quite a number of necessary socioeconomic variables cannot be maintained after that time point. Meanwhile, no publicly published county-level socioeconomic dataset could be obtained as an alternative data resource for replacing or filling in those missing variables. Therefore, in this study, we focused on the historical county-level areal data in southwest China spanning a ten-year period from 2002 to 2011 in order to ensure that more representative socioeconomic factors could be added for this long-term analysis.

The environmental climate data were obtained from the National Meteorological Information Center (http://data.cma.cn/) [30]. In contrast, the other types of environmental factors were extracted from the Resource and Environmental Data Cloud Platform (http://www.resdc.cn/), which contained multiple aspects including geography, topography, hydrology, vegetative cover, transportation, remote sensing index, et cetera. Since the environmental variables, including climate, river density, elevation, road network density, and slope, had no variations at the time scale, they were only added into the spatial nonstationary analysis as part of the local-scaled modeling.

The descriptive statistics of the above experimental data, including both the response variable and 32 potential influencing variables, are summarized in Appendix A (Table A1). Statistical results revealed that most of the variables did not fit the normal distribution, and the dimensional differences arising from the data unit among different variables demonstrated significant variations. Thus, we firstly conducted a logarithmic transformation for the response variable, in other words, a prior log-Gaussian likelihood function, to approximate a normal distribution for Bayesian hierarchical regression modeling. Furthermore, all the covariates were standardized via the adoption of the z-score method into dimensionless values to be fairly compared within the same framework.

### 2.2. Covariates Screening Methods

Two well-developed and commonly adopted methods, namely the multicollinearity evaluation and random forest [31], were employed to select the most important explanatory variables from the 32 potential socioeconomic and environmental factors and were further added into the regression modeling. First, the variance inflation factor (VIF) was calculated as the indicator for measuring multicollinearity, which implied the existence of a correlation between explanatory variables [32]. The empirical judgment suggests that 0 < VIF < 10 indicates that the multicollinearity is slight and acceptable [33], 10 ≤ VIF < 100 indicates relatively strong multicollinearity, while VIF ≥ 100 indicates severe multicollinearity [30]. In this case, a potential variable with a VIF value greater than 5, which is a stricter standard, was firstly removed.

Following the VIF-based step, the random forest, an integrated machine learning method based on the decision tree, was adopted in order to further screen the explanatory variables by calculating another two indicators, which respectively represented a factor’s relative importance and contribution to the model fitness [31]. The first screening indicator is the mean decrease impurity (MDI) that quantifies the change of Gini impurity (information gain), which is usually used to measure the information uncertainty or the degree of the confusion system [34]. The second screening indicator is the mean decrease accuracy (MDA), of which the main idea is to disrupt the order of the eigenvalues of each feature and then to measure the effect of order change on the model accuracy. For each potential variable, higher values of both MDI and MDA indicated the increased significance of this variable in terms of improving the overall model performances. Following the screening outcomes of VIF, a list of socioeconomic and environmental variables with higher values of both MDI and MDA were finally selected for the next-step regression modeling.

### 2.3. Local Spatiotemporal Regression

#### 2.3.1. Bayesian STVC Model

Spatiotemporally varying coefficients (STVC) model, a Bayesian-based local spatiotemporal regression approach, has been proposed for space–time big data with the core mission of detecting both the spatial and temporal heterogeneous relationships between the response and different covariates variables, via estimating posterior local-scale regression coefficients across space and over time [28,29]. Compared with the global-scale spatiotemporal regression models, the fundamental advantage of the local-scale Bayesian STVC model is further incorporating the spatial-temporal autocorrelated nonstationarity for the observable underlying covariates within the Bayesian hierarchical modeling (BHM) framework, such as the socioeconomic and environmental factors concerned in this case.

Herein, the top-level of a simplified interpretation-driven type of Bayesian STVC model for estimating the county-level hospital beds number per ten thousand people (Y) is presented in Equation (1).

(1) $$\eta_{it}=g(Y_{it})=\beta_{0}+\sum_{n=1}^{N}f(\mu_{n,i}SE_{n,it})+\sum_{n=1}^{N}f(\gamma_{n,t}SE_{n,it})+\sum_{k=1}^{K}f(\mu_{k,i}EX_{k,it})+\sum_{k=1}^{K}f(\gamma_{k,t}EX_{k,it})$$

where $\eta_{it}$ is the structured additive linear predictor, $Y_{it}$ is the observed values of the county-level healthcare resources in space *i* and time *t*, in which *I* = 1, …, *I* (*I* = 461) represents the county-level areal unit, and *t* = 1, …, *T* (*T* = 10) indicates the temporal points among ten years. g(·) is the likelihood function to link $Y_{it}$ and $\eta_{it}$, which follows a log-Gaussian prior data distribution herein. $SE_{n,it}$ denotes the observed values of N socioeconomic factors in the *i*-th county for the *t*-th year, and $EX_{k,it}$ denotes the observed values of K environmental factors in each county-year unit.

The posterior estimated parameters from Equation (1) include five components, among which $\beta_{0}$ is the global-scale intercept fixed effect, $\mu_{n,i}$ and $\gamma_{n,t}$ are the local-scale spatial and temporal coefficients for the N socioeconomic covariates, while $\mu_{k,i}$ and $\gamma_{k,t}$ are the local-scale spatial and temporal coefficients for the K environmental covariates. In particular, $\mu_{n,i}$ and $\mu_{k,i}^{}$ are called space-coefficients (SC) that represent the spatially heterogeneous response-covariate relationships within each area. $\gamma_{n,t}$ and $\gamma_{k,t}^{}$ are called time-coefficients (TC), representing the temporally heterogeneous response-covariate relationships in each time point.

Function *f* () represents the sub-level latent Gaussian models (LGMs) to fit the spatial and temporal nonstationary random effects for estimating the local-scale SC and TC of each covariate [35,36].

For the spatial LGM, we assume that the random effects of spatial structural variability (spatial autocorrelation/dependence) follow an intrinsic conditional autoregressive (CAR) prior [37], which is formulated using Equation (2).

(2) $$\mu_{i}|\mu_{j\neq i}\sim N(\frac{1}{m_{i}}\sum_{i\sim j}^{}\mu_{i},\frac{\sigma^{2}}{m_{i}})$$

where *i~j* represents that county *i* and county *j* are adjacent to each other, *m_i_* represents the number of counties sharing the boundary with the *i*-th county, and $\sigma^{2}$ represents the variance components [38]. The CAR prior model assumes that the healthcare-covariate relationship in a county is geographically autocorrelated with surrounding neighbor counties. In the top-level of the STVC formula in Equation (1), the CAR prior is applied to both socioeconomic and environmental covariates, expressed as $f(\mu_{k,i}EX_{k,it})$ and $f(\mu_{n,i}SE_{n,it})$, respectively.

For the temporal LGM, the random walk (RW) model is adopted as a prior sub-level model to fit the temporal structural random effects through adjacent dynamic modeling [39], whose prior density π is formulated with Equation (3).

(3) $$\pi (\gamma_{t}|\sigma_{\gamma }^{2})\propto \exp(-\frac{1}{2\sigma_{\gamma }^{2}}\sum_{t=2}^{T}{\left(\gamma_{t}-\gamma_{t-1}\right)}^{2})$$

The RW prior model assumes that the temporal variation of the healthcare-covariate relationship is affected by the adjacent time points (temporal autocorrelation), of which the time trend could be expressed as a smooth linear or nonlinear curve. Like the settings of the spatial LGM for Equation (1), the RW model is also utilized for both socioeconomic and environmental covariates, in other words, $f(\gamma_{n,t}SE_{n,it})$ and $f(\gamma_{k,t}EX_{k,it})$.

#### 2.3.2. Models Implementation

As a comprehensive evaluation, we compared the Bayesian STVC model (model 5) with the other four benchmark regression models (models 1–4) to verify its superiorities. To be specific, model 1 and model 2 were global regressions that could only fit for stationary variable relationships, between which the difference was that model 2 additionally incorporated the spatiotemporal random effects for intercepts. In contrast, models 3 and 4 belonged to the local regression family for detecting nonstationary response-covariate relationships. Among three local regression models, model 3 and model 4 were two special cases of an STVC model (model 5), aiming to test the necessity of simultaneous incorporation of both spatial and temporal random effects for the nonstationarity within covariates.

Model 1 was an ordinary multivariate regression model, formulated in Equation (4).

(4) $$\eta_{it}=\beta_{0}+\sum_{n=1}^{N}\beta_{n}SE_{n}+\sum_{k=1}^{K}\beta_{k}EX_{k}$$

where $\beta_{0}$ is the intercept term, $\beta_{n}$ and $\beta_{k}$ are the global-scale coefficients of the stationary healthcare-covariate relationships for socioeconomic and environmental covariates, respectively.

Model 2 was a general spatiotemporal multivariate regression model, as presented in Equation (5). Compared with model 1, model 2 further incorporated the spatial and temporal random effects of intercepts, which represent the smoothed variations of the response variable itself.

(5) $$\eta_{it}=\beta_{0}+\sum_{n=1}^{N}\beta_{n}SE_{n}+\sum_{k=1}^{K}\beta_{k}EX_{k}+f(\xi_{i})+f(\psi_{t})$$

where $\xi_{i}$ is the space-intercept (SI), and $\psi_{t}$ is the time-intercept (TI). Following standard settings for SC/TC and the mainstream spatiotemporal models, we also employ the same LGMs, namely, CAR and RW prior sub-level models, to estimate the posterior SI and TI herein [36,39,40].

Model 3 is a temporally varying coefficients (TVC) model only concerning the temporal nonstationary random effects for socioeconomic and environmental covariates, as formulated in Equation (6).

(6) $$\eta_{it}=\beta_{0}+\sum_{n=1}^{N}f(\gamma_{n,t}SE_{n,it})+\sum_{k=1}^{K}f(\gamma_{k,t}EX_{k,it})$$

Model 4 is a spatially varying coefficients (SVC) model only accounting for the spatial nonstationary random effects in covariates of socioeconomic and environmental factors, as shown in Equation (7).

(7) $$\eta_{it}=\beta_{0}+\sum_{n=1}^{N}f(\mu_{n,i}SE_{n,it})+\sum_{k=1}^{K}f(\mu_{k,i}EX_{k,it})$$

Model 5 is a customized Bayesian STVC model designed for this empirical case, which has been fully introduced in Equations (1)–(3). Herein, model 5 is a simplified version of a general STVC model by removing the global-scale stationary fixed effect of auxiliary covariates (same as in model 1), as well as the spatiotemporal random effects of intercepts (same as in model 2). These interpretation-driven STVC settings make sense particularly for exploring the response-covariate relations along with explicit spatial patterns and temporal trends, aiming at explaining the mechanism behind the research object, as it removes the potential interactive influences of local intercepts [29].

#### 2.3.3. Bayesian Inference and Model Evaluation

In this work, we developed five regression models above under the flexible BHM framework using R software, and the integrated nested Laplace approximation (INLA) [41] computational approach was employed as the Bayesian inference method to estimate the posterior parameters due to its relatively short computation time with accurate estimation [42]. In terms of a BHM-based STVC model, there were three levels, with each level containing several further sub-levels. For the first data distribution level, we utilized the log-Gaussian likelihood function. For the second space–time process level, we combined the sub-level LGM models, including CAR and RW, to consider the spatial and temporal random effects in particular for those vital explanatory covariates with nonstationary assumption [28]. For the third parameter level, we specified the non-informative priors for the parameters and their variance components, so that the observed space–time data could have the most significant impact on the posterior distributions [43,44].

We further evaluated the performances of each Bayesian-based regression model in four aspects, namely model fitness, complexity, predictive capacity, and explained variation [39,45]. First, the deviation information criterion (DIC) [46] and the Watanabe–Akaike information criterion (WAIC) are two widely used indices that describe Bayesian model fitness. Two indices of model fitness are the smaller, the better. Second, the model complexity is quantified by two indices of effective parameters (*P_DIC_* and *P_WAIC_*) that can be obtained with both DIC and WAIC methods synchronously. Two indices of model complexity are the smaller, the better. Third, the model predictive power is quantified by a logarithmic score (LS) that is retrieved from the conditional predictive ordinates under a leave-one-out cross-validation. LS is also considered as the smaller, the better [47]. Lastly, the coefficient of determination (*R*^2^) is commonly used in regression models for evaluating the explained variation or variance, which is defined as the degree to which predicted and actual values are consistent. A higher value of *R*^2^ indicates a greater variation that the model can explain [22].

## 3. Results

### 3.1. Covariates Selection

Considering the multicollinearity issue and the relative importance of each potential variable, ten factors were selected as the key explanatory variables for modeling out of 32 potential socioeconomic and environmental variables, which was conducted based on two progressive screening steps.

To be more specific, in the first step, the VIF values of the two types of factors were calculated respectively and summarized in Appendix A (Table A2). We removed variables with higher multicollinearity by setting five as the VIF screening threshold value. Generally, different factors representing the socioeconomic level possess multicollinearity, and one convenient way to deal with this issue is to choose the ones that are the most representative (with the smallest VIF) instead of selecting all the factors. This method can also be applied to the selection of environmental variables. By setting VIF < 5 as the screening benchmark, we retained six factors for the socioeconomic aspect, namely, SE6, SE10, SE17, SE18, SE19, and SE20. Similarly, seven factors were retained for the environmental aspect, namely, EX1, EX2, EX3, EX6, EX9, EX11, and EX12.

**Figure 2:**
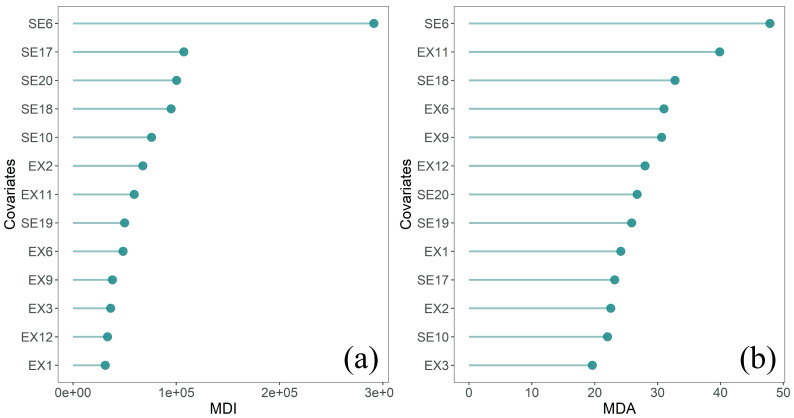
Variables’ relative importance evaluated by random forest-based indicators: (**a**) mean decrease impurity (MDI) and (**b**) mean decrease accuracy (MDA).

In the second step, based on the remaining factors from the first step, we further evaluated the relative importance (contribution) of each factor using MDI and MDA indicators that were obtained from the random forest method, as illustrated in Figure 2. We chose those factors with higher contributions, by retaining the first half percent (seven) factors for both evaluation indicators MDA and MDI, among which some factors were ranked as the top seven most indicative factors in terms of two aspects. After this screening step, a total of ten explanatory variables including five socioeconomic factors (i.e., SE6, SE10, SE17, SE18, and SE20) and five environmental factors (i.e., EX2, EX6, EX9, EX11, and EX12) were selected as the core covariates (renamed as X1–X10 in Table 3) and added into the next-step serial regression modeling.

### 3.2. Model Evaluation and Comparison

Table 2 summarizes the performances of the five comparative Bayesian regression models based on comprehensive consideration of six selection criteria statistics. Model 5 (STVC) was proven to be the optimal Bayesian-based model with the smallest values of DIC, WAIC, and LS. The indicator *R*^2^ further revealed that model 5 also had the highest model-explained variation (92%), which met an essential decision requirement for the following influencing factors analysis. Moreover, model 5 surpassed both model 3 (TVC) and model 4 (SVC), thus indicating the necessity of incorporating both the spatial and temporal random effects in fitting the nonstationary variables relationships. However, following *P_DIC_* and *P_WAIC_*, we noticed that model 5 had a drawback with a much higher complexity than the mainstream benchmark models 1 and 2. For instance, the complexity (*P_DIC_*) of model 5 was about 96 times higher than that of an ordinary multivariate regression (model 1), and even 2.5 times higher than that of a general spatiotemporal multivariate regression (model 2).

In spite of the high complexity embedded in the STVC model, the necessity of increasing such complexity should be highlighted as indispensable mainly due to reasons as below. First, the STVC model surpassed all the other four regressions concerning model fitness, predictive power, and explained variation. Among them, model 2 is a popular spatiotemporal regression model that typically demonstrates higher prediction accuracy. The increase in prediction accuracy of model 2 is attributed to the core incorporation of the spatiotemporal random effects for intercepts. However, such spatiotemporal intercepts lack the capacity for interpretation, that is, it is impossible to explain how different observable explanatory factors affect the target variable of interest at the local space–time scale exactly. In contrast, it should be noted that only the STVC model had the capacity for synchronously detecting the spatial and temporal heterogeneous relationships between variables for further interpretation and inference. What is worth mentioning is the customized model 5 for this case still achieved the best model performance, even without taking into account the spatiotemporal random effects of intercepts. Hence, we chose the real interpretive Bayesian STVC model, with the best model performance, as the final regression for conducting further analysis of spatiotemporal influencing factors as well as the estimation of spatiotemporal inequality maps of healthcare resources.

### 3.3. Covariates’ Global Scale Impacts on Healthcare Resources

Table 3 summarizes the posterior parameters of the selected socioeconomic and environmental covariates (X1–X10), including the global-scale coefficient representing the stationary variable relationship, standard deviation (SD), as well as credible intervals (CIs), which were estimated by an ordinary multivariate regression model (model 1). As all the models adopted followed a log-Gaussian prior data distribution, and all the covariates were standardized, the model-estimated coefficients were not under the normal distribution scale or with the original unit. Thus, a reasonable explanation for these coefficients was that they represented the relative contribution (impacts) of covariates on the healthcare resources outcomes.

The overall coefficients of ten covariates were greater than zero, indicating that both socioeconomic and environmental factors were positively contributing to the development of hospital beds at a global scale in southwest China. For the five socioeconomic covariates, X1 and X5 reflected the personal economic condition based on an individual or family, X2 and X3 represented the regional economic condition based on the government and macro-control, and X4 represented the population condition. The other five environmental covariates covered two aspects. X6, based on nightlight satellite remote sensing data, indicated the county-specific urbanization level. Climate (X7), transportation (X8 and X10), and topography (X9) reflected a general geographical situation. Particularly, the residents’ saving deposits per capita (X1) was the most significant socioeconomic factor with a much higher positive contribution among the ten covariates, followed by environmental factors X6, X7, and X9.

As indicated by CIs, most of the covariates demonstrated lower uncertainties and acceptable statistical significance, except for covariates X4 and X5, of which the posterior probability density functions had a zero value between the lower and upper CIs. However, these stationary assumption-based global-scaled results might not be suitable for a finer-scaled space–time dataset. As shown in Table 2, model 1 could only explain 75% variation of the response variable (*R*^2^ = 0.75), while model 5 could explain a much higher variation of 92% (*R*^2^ = 0.92) via the adoption of the same ten covariates under a spatiotemporal nonstationary assumption. Based on all these considerations, we finally added all the ten covariates into the other types of modeling (models 2–5), which were also proven to possess higher contributions as indicated by the random forest method.

### 3.4. Covariates’ Temporal Heterogeneous Impacts on Healthcare Resources

**Figure 3:**
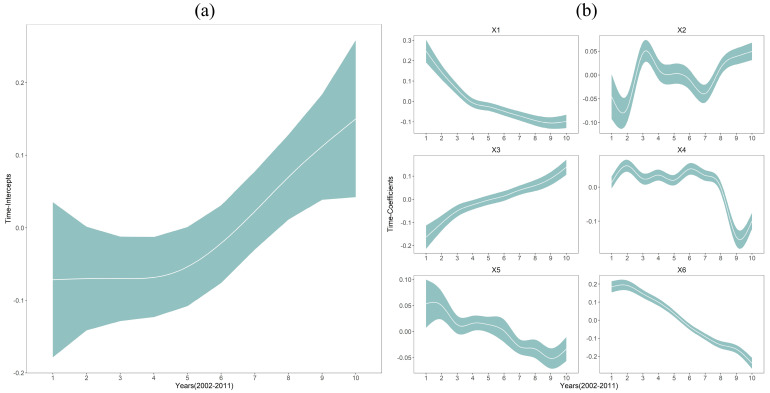
(**a**) Time-intercepts (TI) plot on behalf of the crude temporal variation of hospital beds in southwest China during 2002–2011, and (**b**) time-coefficients (TC) plots representing the temporal heterogeneous hospital bed-covariates relationships: X1, residents’ saving deposits per capita; X2, total investment in fixed assets per capita; X3, GDP per capita; X4, urban worker population density; X5, total retail sales of consumer goods per capita; and X6, nighttime light index.

In Figure 3, we presented a TI plot (a) and six TC plots (b) to represent the crude temporal trend of hospital beds and the temporal heterogeneous hospital bed-covariates relationships, which were estimated by model 2 and model 5, respectively. From Figure 3a, we found that the hospital bed level over the entire southwest China showed an increasing trend year by year, with little change for the first five years, but a noticeable upward trend for the last five years. The TI plot indicated that the healthcare resources of hospital beds in southwest China have dramatically developed since 2006.

Furthermore, from Figure 3b, we found that the temporal hospital bed-covariates relationships were not consistent, but with diverse nonlinear variations over 2002–2011. Unlike the global-scale coefficients listed in Table 3, the TC plots estimated by a Bayesian STVC model were able to visualize local-scale nonstationary regression relationships over periods. Generally, we discovered that the impacts of personal economy, which was reflected by the residents’ saving deposits (X1) and the total retail sales of consumer goods per capita (X5), were diminished from 2002 to 2011. In contrast, the impacts of the government-led regional economy, which was depicted by the total investment in fixed assets (X2) and the GDP per capita (X3), became more energetic during the corresponding period, while all the other covariates showed downward trends. Furthermore, we noticed that after 2011, X2, X3, and X4 showed a relatively clear upward trend, suggesting that the key to further promoting the overall hospital bed-levels in southwest China might be focused on these socioeconomic conditions. These findings also indicated that the dominant factors affecting hospital beds of southwest China had changed during the studied decade. 

### 3.5. Covariates’ Spatial Heterogeneous Impacts on Healthcare Resources

**Figure 4:**
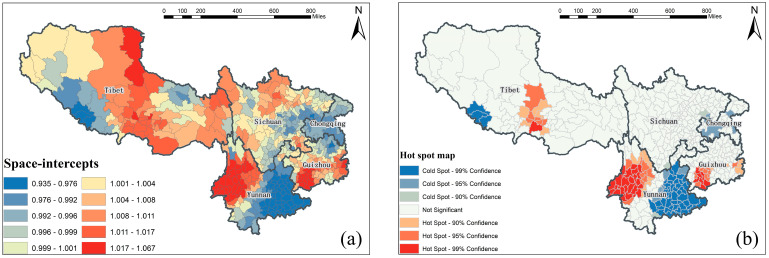
(**a**) Space-intercepts (SI) map representing the crude geographical distribution of the county-level hospital beds across southwest China, and (**b**) SI’s clustered hot spot map.

**Figure 5:**
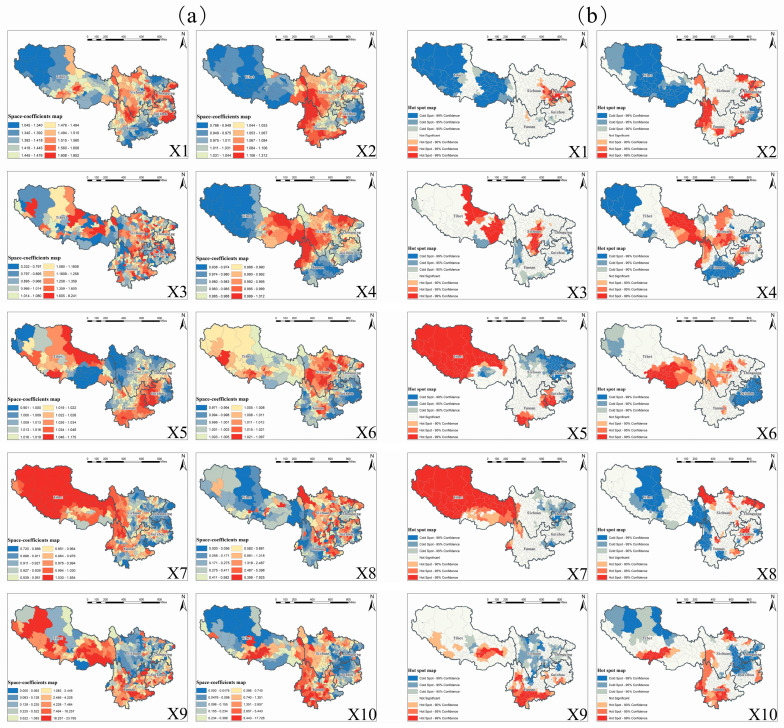
(**a**) Space-coefficient (SC) maps for detecting the spatially heterogeneous hospital bed-covariates relationships of both socioeconomic and environmental factors at the county level across southwest China, and (**b**) SC’s clustered hot spot maps: X1 residents’ saving deposits per capita; X2, total investment in fixed assets per capita; X3, GDP per capita; X4, urban worker population density; X5, total retail sales of consumer goods per capita; X6, nighttime light index; X7, wind speed; X8, river network density; X9, slope; X10, road network density.

Spatially, we firstly retrieved the SI map and its clustered hot spot map with model 2, to detect the in situ autocorrelated geographical distribution of the county-level hospital bed-levels across southwest China, as presented in Figure 4a,b respectively. Then we produced the SC maps and their clustered hot spot maps, as presented in Figure 5a,b to further explore the spatially heterogeneous impacts of different socioeconomic and environmental covariates on hospital bed outcomes at the county level by using model 5. It should be noted that the “Not Significant” regions in the hotspot maps below, including Figure 4b and Figure 5b, did not indicate the absence of impacts. However, the impacts within these regions were not statistically significant enough to form an agglomeration.

Moreover, in Figure 5, we detected significant spatial clusters in all hospital bed-covariates relationships for both socioeconomic and environmental factors, suggesting the necessity of incorporating the spatial autocorrelation to fit nonstationarity within an STVC modeling. In practice, regarding an influencing covariate of interest, we could visually distinguish which local-scale areas were sensitive to this covariate for improving healthcare resources, as well as which areas were not, by directly applying the target SC map of that covariate. Furthermore, within each county area, we could give a specific county-level policy proposal about the relative impacts of the ten different socioeconomic and environmental covariates on the local-scaled hospital beds outcome, by vertically integrating the local-scale county-level information via all the SC maps together [29].

Apart from the spatially county-level hospital bed-covariates relationships detected from Figure 5a, we further summarized the province-level clustering results from SC’s hot spot maps in Figure 5b. For example, in Sichuan province, the regional economy (X3), employment level (X4), and urbanization level (X6) jointly had spatially positive clustered impacts on hospital beds outcomes, especially in the central region of Sichuan. In contrast, in Chongqing municipality, personal economy (X1) and economic investment (X2) played significant positive roles in improving hospital beds over the entire study area. Such spatially positive clustered influences of different factors were characterized by strong geospatial heterogeneity in Guizhou province, where the employment level (X4) and river network density (X8) were found to be the main positive determinants in the central region. At the same time, the residents’ consumption-ability (X5) remained a critical problem in western counties. In Yunnan province, the economic investment (X2), topography (X9), and road network density (X10) were general positive contributors to hospital bed outcomes at a regionally clustered scale. In Tibet, the largest region in southwest China, hospital beds were positively affected by multiple primary socioeconomic conditions, including the regional economy (X3), employment level (X4), and residents’ consumption-ability (X5). Moreover, the unique natural environment in Tibet might have potentially contributed to the phenomenon where a list of environmental factors such as urbanization level (X6), topography (X9), and road network density (X10) had exceptionally positive impacts on hospital bed distributions in certain regions.

### 3.6. Estimated Spatiotemporal Maps of Healthcare Resources Equalities

**Figure 6:**
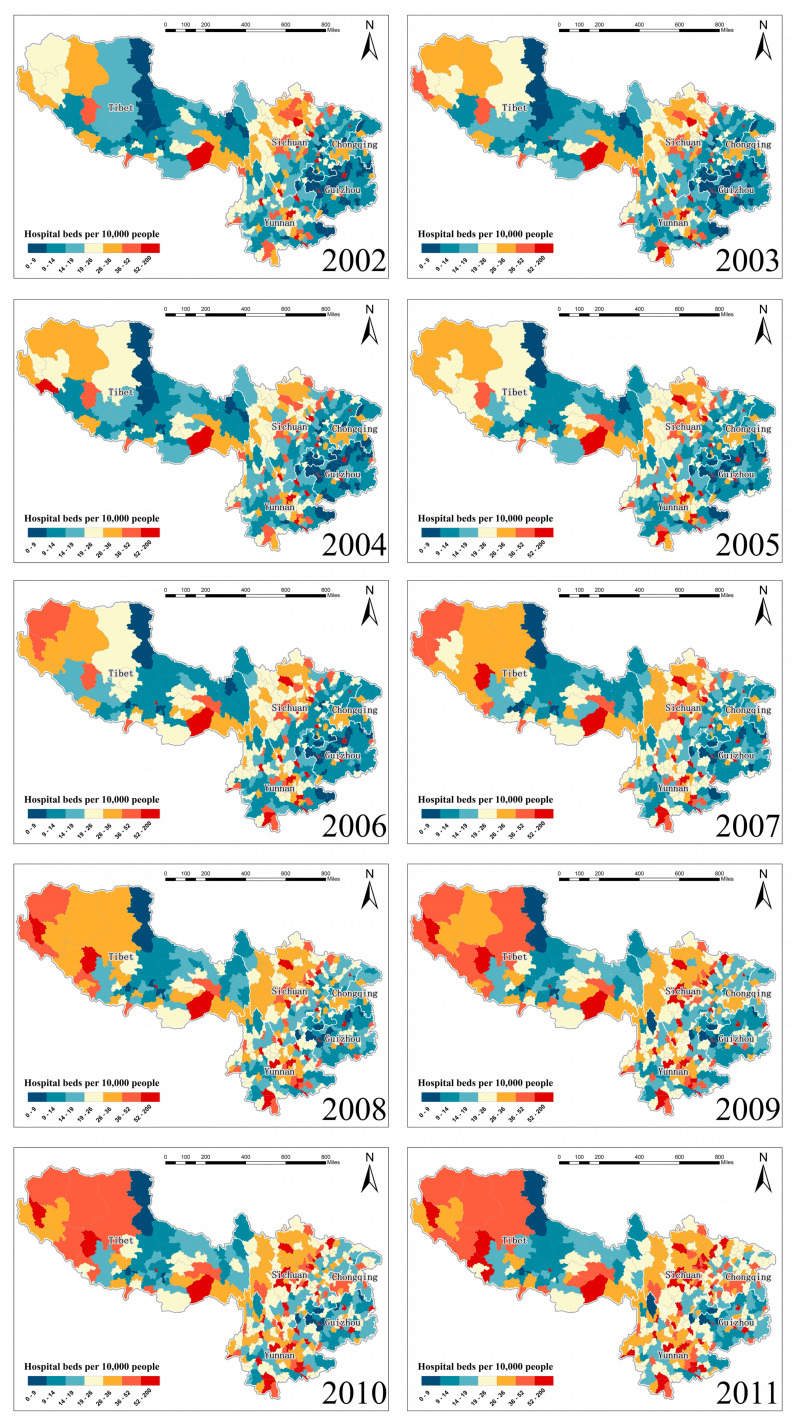
Estimated spatiotemporal equalities maps of the county-level healthcare resources of hospital beds across southwest China from 2002 to 2011.

Finally, we produced the first series of complete county-level healthcare resources equalities maps (Figure 6) using the hospital bed number per ten thousand people as the proxy indicator over southwest China during 2002–2011 based on the adoption of the optimal Bayesian STVC model (model 5). The newly model-estimated hospital bed equality maps could offer people with both higher (e.g., in situ counties with missing values) and more intuitive (e.g., smooth the county-level extreme outliers) information for accurate optimization of healthcare resources at the local scale. In general, we found an overall enhancement in terms of the situation of hospital beds in southwest China over the studied time period. Especially after 2005, the hospital bed resource of southwest China was significantly improved both regionally and locally, of which the visualization discovery was consistent with the findings from the TI map in Figure 3a. In addition, we found that different regions demonstrated diverse improvement intensity. For instance, most of the blue-colored counties in Yunnan province in 2002 were shifting to yellow/red color over the illustrated ten years, suggesting that the hospital bed improvement of Yunnan province was the most significant, compared with all the other provinces. Moreover, we noticed that despite the county-level hospital bed levels demonstrated overall enhancement by changing from blue color to yellow/red color at the local scale from 2002 to 2011; several low-healthcare-level counties still remained in blue color in the latest year, which were mainly distributed in Guizhou, Tibet, and Yunnan provinces.

## 4. Discussion

The socioeconomic status, involving the personal economy, government-led regional economy, and population, was verified as a critical aspect affecting hospital bed outcomes, which was found to be consistent with previous studies. For instance, Qin et al. proved that the regional economy and population had an essential impact on the convergence pattern and the convergence speed in the geographic distribution of healthcare resources in China [19]. Furthermore, Pan et al. reported a strong positive correlation between the economy and hospital bed concentration at county levels, including both the personal economy and the regional economy [13]. Unemployment rate [21], healthcare expenditure [8,17], and population size [14] were also validated by previous studies as essential socioeconomic factors.

In addition to socioeconomic status, the environmental condition was also identified as an equally influential contributor to hospital bed development, which represented the urbanization progress and a general geographical condition. Among five environmental factors analyzed, slope [8,16] and road network density [8,17] were proven as influencing factors on the geographical distribution of hospital beds. However, river network density, wind speed, and nighttime light index were firstly found to have substantial driving effects on hospital bed differentiation in this case. To the best of our knowledge, this is also the first study in southwest China that evaluated hospital beds inequalities via incorporating both socioeconomic and environmental aspects [13,23].

Beyond identifying critical influencing factors based on the incorporation of both socioeconomic and environmental aspects, a more impressive contribution of this work is that we comprehensively investigated both the global and local spatiotemporal relationships between the county-level disparity distribution of hospital beds as well as a diverse range of influencing factors in southwest China. An innovative aspect inherent in this study was that compared with previous stationary-based studies which were only limited to the investigation on global-scaled socioeconomic factors [48,49,50], we had incorporated both socioeconomic and environmental factors during the statistical analysis procedure based on the adoption of an innovatively developed Bayesian-based local spatiotemporal regression STVC model [28], which served as a potent tool for facilitating the detection of local-scaled spatiotemporal heterogeneous hospital bed-covariates relationships. In the area of public health, this innovative methodology has made our study the first research ever conducted in China to examine the local-scaled spatiotemporal heterogeneous impacts of both socioeconomic and environmental covariates on the healthcare resource equalities in the aspect of hospital bed allocations [11,16,17].

With regards to hospital bed-covariates relationships in a global range, personal economic status was identified as an essential aspect, which was likely induced by the structural characteristics of China’s public–private hybrid financing arrangements [13]. As traditional medical insurance plans were only able to compensate for expensive medical costs partially, a large percentage of total medical expenditures were still covered by out-of-pocket payments. It is not difficult to imagine that the continuous growth of our personal economy will be very likely to improve residents’ ability to afford advanced medical services. Thus, such increased market demand has become a stimulant for the expansion of regional healthcare resources. In addition to personal economic status, the socioeconomic impacts of both government-led regional economy [8,19] and urban worker population [14,21] were also detected, suggesting that the driving force of government inputs and population demands should also not be ignored [14,19]. Furthermore, in terms of the environmental conditions, we discovered that urbanization and the general geographical situation also had positive influences on hospital bed distribution in southwest China. The urbanization indicator of the nighttime light index can be regarded as an environment-based socioeconomic indicator representing the level of the regional economy and population density [51]. Geographical indicators such as road and river network density reflected the degree of traffic development, which could affect the spatial accessibility to healthcare resources [52,53,54]. Unfortunately, such global-scaled findings might not be applicable for studies involving large study areas or long-term analysis, especially via the adoption of a finer-scaled space resolution as we employed in this study [55].

In terms of the temporal development of hospital beds in southwest China, a stable development pace at low development levels was found from 2002 to 2005. However, such development pace started to accelerate continuously from 2006, which was consistent with previous findings [56]. Moreover, regarding temporal hospital bed-covariates heterogeneous relationships, the consideration of the temporal nonstationarity has enabled us to further identify a gradual switch from the personal economy to the government-led regional economy, which served as the time-scale leading factor for affecting the development of healthcare resources, instead of generally addressing personal economy as an essential factor from a global-scaled perspective. The growing impacts of the government in healthcare resources was presumably induced as the result of the first phase (2009–2011) of China’s healthcare reform, during which the expansion of social health insurance coverage was emphasized for benefiting residents nationwide as well as for strengthening nationwide healthcare infrastructure constructions. In sharp contrast with the past two decades during which personal economic status had served as the determinant for both healthcare quality and accessibility, since the initiation of the first phase of China’s healthcare reform, the governmental impact has become an overwhelming factor among healthcare settings, especially in the aspect of providing financial support for improving basic healthcare quality [57].

Spatially, we detected significant geographical clustered regions for both hospital beds and hospital bed-covariates relationships in the form of maps. However, it should be noted that the spatial nonstationarity embedded among variable relationships was further characterized by strong geospatial differentiation for all socioeconomic and environmental covariates at both county and provincial levels [11,12,13]. Practically, within each administrative county area, an area-specific policy can be proposed based on the analysis of the individual impacts of each socioeconomic and environmental covariate on hospital bed outcomes [29]. However, such county-level implications would be too limited to be adopted in a more extensive administrative range. Thus, hot spot maps were further retrieved for discovering regionally clustered hospital bed-covariates relationships in order to further assist in policy-making procedures on a coarser scale, for example, provincial-level. We discovered that the provincial-level hospital bed-covariates relationships among different provinces had strong spatial stratified heterogeneity [58], where a single or multiple socioeconomic or environmental factors played predominant roles in affecting healthcare resource allocations in the aspect of hospital bed distributions. In China, healthcare resources largely depend on local economic development, especially on the financial capacity of local governments. The spatially strong aggregation of healthcare resources also reflected the differences and aggregation of socioeconomic development among administrative regions to some extent. Lessons learned from regional cases could be further optimized and adapted in a nationwide range for policy-making purposes [57]. It should be highlighted that the allocation of healthcare resources should be conducted at central governmental levels from a holistic perspective instead of at lower administrative levels as a strategy for minimizing geo-clustered inequalities induced by region-specific financial resources, as well as for ultimately achieving healthcare resource allocation equalities among different regions [59].

Another contribution of this study was the innovative production of a new series of maps depicting healthcare resource equalities over southwest China at an administrative county level, from 2002 to 2011. The adoption of the local-scaled spatial and temporal heterogeneous hospital bed-covariates relationships detected before has facilitated our estimation of the maps with higher accuracy. Based on the most up-to-date inequality map, a list of counties with the most inadequate hospital bed allocations were mainly identified in Guizhou, Tibet, and Yunnan, which indicated that resource reallocation should be emphasized as the priority in such underdeveloped areas via the implementation of relevant policies and strategies at governmental levels. Our estimation of a series of spatiotemporal healthcare equality maps is expected to assist policymakers in the analysis and implementation procedures of relevant policies from a more direct-viewing perspective, which would further contribute to the optimization of healthcare resource allocations in a more rational manner.

Practically, based on the previous discussions, improving residents’ living standards should be addressed as the key strategy regardless of disparities embedded in different geographical regions or different time slots. Furthermore, our findings are expected to provide evidence-based implications for future government-led policy-making procedures. First, the influence of geographical environment should be emphasized as an indispensable aspect at governmental levels in terms of propelling the development of healthcare resources. In addition, fortifying governmental investment, along with encouraging macroeconomy development, should be emphasized as the essential strategies in the future in order to achieve promoted healthcare resource allocations in the study area of southwest China. In terms of geo-clustered inequalities of healthcare resources, a higher level of integrated health planning might be proposed as a solution for mitigating such a problem to some extent. Financial support needed for facilitating such integrated health planning might be obtained from special funding programs designed for optimizing healthcare resources as part of the central or provincial financing budgets [59]. Last but not least, in the process of large-scale geospatial healthcare assessment, well-rounded consideration incorporating various region-specific factors should be addressed at both spatial and temporal scales in order to achieve the optimization of healthcare resource allocation via the formulation of region-specific policies and strategies, which would serve as indispensable complements to the general policy as well as being further adopted in the other comparative countries or regions.

Lastly, we would like to talk about the applied cutting-edge Bayesian STVC model, in terms of its value in solving a list of complex problems in this empirical study [29]. The STVC model was the first Bayesian-based spatiotemporal local regression approach developed for space–time big data, with a core mission to simultaneously explore the autocorrelated spatial and temporal nonstationarity inherent in variables relationships, by taking advantages of both Bayesian statistics [60] and hierarchical modeling [28]. Such spatiotemporal heterogeneous variables relationships cannot be detected via the adoption of conventional global-scaled regressions [44], local-scaled spatial or temporal regressions [61,62], or the mainstream spatiotemporal regressions [39]. The local-scaled outputs from an interpretation-driven STVC model included three main components, namely, the space-coefficients (SC) maps, the time-coefficients (TC) plots for nonstationary covariates, and the estimated complete spatiotemporal maps for the target variable of interest. All these deliverables were expected to assist the governmental formulation of multi-leveled policies and strategies in both spatial and temporal dimensions, which should be highlighted as innovative progress compared with previous studies in this area [13,14,16]. Specifically, the separate provision of SC maps and TC plots for each covariate as the direct outputs of the Bayesian STVC model has great potential in facilitating stakeholders to immediately figure out the spatial and temporal autocorrelated regularities embedded in variable relationships, thus exempting the necessity of further processing the newly estimated space–time coefficients [26,27]. In addition, regardless of the missing values inherent in the original response variable of interest, the Bayesian STVC model can still provide an estimation for a complete series of SC maps and TC plots in order to demonstrate constantly changing relationships among different variables. Such convenient estimation had been achieved with the help of the prior spatial/temporal latent Gaussian models within the BHM framework [29]. Furthermore, the simplified interpretation-driven type of Bayesian STVC modeling for this case still outperformed the mainstream regression models, significantly enhancing the model applicability and expandability for the prediction purpose.

Apart from all these achievements aforementioned, some aspects should be addressed as the limitations of this work. As a couple of environmental factors lacked variations at the temporal scale, the applied methods were only able to detect the spatial nonstationarity impacts of these factors. The most up-to-date satellite remote sensing technology could be applied as a potential solution for tackling such data deficiencies. Another limitation inherent in this study was that the dataset we adopted for analysis failed to provide the most up-to-date information, due to the later revision of China’s county-level statistical yearbooks during which multiple vital variables were removed, and several new variables were added [22]. Failing to find a solution to this problem, in this study, we had to sacrifice the timeliness of our research in order to keep the space scale to the minimum county level, as well as to ensure the comprehensiveness of socioeconomic factors. Despite such flaws embedded in our data, the productive results from Bayesian STVC modeling provided us with the first historical retrospective experience for further policy formulation and practical research as an inspiring outcome. Future studies might remain focused on finding solutions for persistent issues related to healthcare resources in China over extended periods, which could be facilitated via the incorporation of increased explanatory variables available [59], as well as the development of more sophisticated Bayesian STVC-based statistical models for producing more sound outputs [29].

## 5. Conclusions

In this study, we validated the hypothetical theories previously proposed that both environmental and socioeconomic aspects had pivotal roles in affecting the small-area healthcare resource inequalities, and such covariate impacts varied locally (heterogeneity) along both space and time scales, which had been supported by an advanced Bayesian STVC modeling of the county-level hospital beds data in southwest China. Practically, within the study area, both temporal nonlinear and spatial clustered healthcare-covariates relationships were found to be significant, and the first series of complete spatiotemporal maps on hospital bed equalities in southwest China spanning ten years was innovatively produced. These empirical findings were consistent with our hypothetical theories, which are all expected to further support the spatiotemporal attribution of healthcare resource inequalities over the entire mainland China in the future lines of research.

Promoting geographical equalities embedded in healthcare resources serves as an essential target to be achieved as part of China’s ongoing healthcare reform, for which spatiotemporal perspective should be constantly emphasized at governmental levels throughout policy-making procedures in order to identify healthcare priorities from a geospatial perspective via addressing varied impacts of both socioeconomic and environmental factors. As a one-size-fits-all policy rarely serves as an effective strategy to be adopted in large countries like China [57], it is highly recommended that geospatial heterogeneity be considered throughout policy-making procedures at regional levels in order to facilitate the formulation and implementation of region-specific strategies for optimization purposes. In order to achieve such region-specific optimization, it is highly recommended that experts from interdisciplinary backgrounds are extensively engaged in policy-making procedures for various healthcare settings as part of the healthcare reform, including public health professionals, health and medical geographers, geographic information science (GIS) practitioners, environmentalists, and spatial statisticians [63,64]. More importantly, the adoption of an innovatively developed Bayesian-based local spatiotemporal regression STVC model in our study demonstrated great value in the spatiotemporal analysis of various influencing factors in the field of public health, which could be further extended for adoption in broader areas such as environment and earth sciences.

## Figures and Tables

**Table 1:** Indicator system of socioeconomic and environmental variables potentially affecting county-level hospital bed resources in southwest China (SE1–SE20 denote socioeconomic variables, and EX1–EX12 denote environmental variables).

Abbreviation	Variables	Units
SE1	Population density	Person/km^2^
SE2	Employee population density	Person/km^2^
SE3	Local telephone users’ density	Person/km^2^
SE4	Local government budgetary expenditures per capita	Yuan
SE5	Local general budget revenue per capita	Yuan
SE6	Residents’ saving deposits per capita	Yuan
SE7	Loan balance of financial institutions per capita	Yuan
SE8	Above-scale total industrial density	Number/km^2^
SE9	Above-scale total industrial output value per capita	Yuan
SE10	Total investment in fixed assets per capita	Yuan
SE11	Junior high school student density	Person/km^2^
SE12	Primary school student density	Person/km^2^
SE13	Gross domestic product (GDP)	Million
SE14	First industry output per capita	Yuan
SE15	Second industry output per capita	Yuan
SE16	Tertiary industry output per capita	Yuan
SE17	GDP per capita	Yuan
SE18	Urban worker population density	Person/km^2^
SE19	Average wage of employees in urban units	Yuan
SE20	Total retail sales of consumer goods per capita	Yuan
EX1	Normalized vegetation index (NDVI)	/
EX2	Nighttime light index	/
EX3	Precipitation	0.1 mm
EX4	Temperature	0.1 centigrade
EX5	Air pressure	1 N/m^2^
EX6	Wind speed	m/s
EX7	Vapor pressure	hPa
EX8	Sunshine hours	hours
EX9	River network density	km/km^2^
EX10	Elevation	Meter
EX11	Slope	°
EX12	Road network density	km/km^2^

**Table 2:** Evaluations for the five alternative regressions considering model fitness, complexity, predictive power, and explained variation.

Index	DIC	WAIC	*P_DIC_*	*P_WAIC_*	LS	*R* ^2^
Model 1	6028.53	6119.66	12.16	84.16	0.68	0.75
Model 2	1928.71	1998.96	475.17	486.15	0.20	0.89
Model 3	7917.86	7934.69	44.59	56.22	0.88	0.51
Model 4	2036.76	2010.19	1144.72	931.87	0.22	0.86
Model 5	1778.38	1749.55	1165.08	944.15	0.19	0.92

Models 1–5: Bayesian-based regression models of ordinary multivariate, spatiotemporal multivariate, TVC, SVC, and STVC; DIC: deviance information criterion; WAIC: Watanabe–Akaike information criterion; *P_DIC_*: effective number of parameters from DIC; *P_WAIC_*: effective number of parameters from WAIC; LS: logarithmic score; *R*^2^: coefficient of determination.

**Table 3:** Global-scale regression statistics of socioeconomic and environmental covariates affecting healthcare resources of hospital beds over southwest China.

Covariate	Name	Coefficient	SD	2.5% CI	97.5% CI
X1	Residents’ saving deposits per capita	0.2159	0.0115	0.1932	0.2385
X2	Total investment in fixed assets per capita	0.0387	0.0088	0.0213	0.056
X3	GDP per capita	0.0499	0.0081	0.0338	0.0659
X4	Urban worker population density	0.0187	0.0099	−0.0009	0.0382
X5	Total retail sales of consumer goods per capita	0.0179	0.0113	−0.0043	0.0401
X6	Nighttime light index	0.0686	0.0132	0.0425	0.0946
X7	Wind speed	0.0778	0.0074	0.0632	0.0923
X8	River network density	0.0337	0.0088	0.0163	0.0509
X9	Slope	0.0954	0.0082	0.0793	0.1115
X10	Road network density	0.0235	0.0084	0.0069	0.0401

## Appendix A

**Table A1:** Descriptive statistics of the modeling data.

Variables	Minimum	Maximum	Mean	Std. Deviation	Skewness	Kurtosis
Y	1.20	135.60	22.87	15.09	2.08	6.00
SE1	0.00001	0.2513	0.0228	0.0267	2.33	9.33
SE2	0.0002	684.30	14.69	36.40	7.85	90.56
SE3	0.0001	1527.90	33.40	82.65	9.88	141.10
SE4	4.99	6,329,247	32,533	194,713	19.74	486.90
SE5	5.67	10,310,602	89,787	302,255	18.75	464.19
SE6	0.75	201,423	5514	8153	8.35	131.73
SE7	4.00	122,519,466	502,703	4,014,174.76	20.24	501.36
SE8	0.51	4558	42	166	16.96	363.20
SE9	1.57	101,037,587	506,231	2,773,609	21.03	585.25
SE10	0.77	256,720	5208	8618.07	8.33	172.10
SE11	0.0002	140.52	12.77	15.47	2.36	9.04
SE12	0.0002	179.10	19.27	20.76	2.24	9.23
SE13	5.26	77,489,300	521,682	2,315,996	19.46	480.79
SE14	4.53	442,463,903	362,658	7,002,860	55.50	3454.81
SE15	3.13	4,366,522,055	2,906,142	74,407,402	49.34	2715.86
SE16	2.75	2,940,718,935	3,408,027	60,985,621	41.88	1872.98
SE17	4.72	617,811	9492	12,017	29.03	1426.97
SE18	0.0001	653.65	15.64	34.12	7.95	98.80
SE19	0.31	32,916	1027	1472	5.18	61.29
SE20	122	728,600	4328	14,171	31.57	1502.91
EX1	0.0844	0.87	0.68	0.16	−1.93	2.85
EX2	0.0001	38.03	1.67	3.11	4.72	31.53
EX3	2093	21,169	10,234	2904	0.05	0.25
EX4	−46.83	224.05	130.81	61.98	−1.11	−0.10
EX5	572.19	977.84	840.22	108.95	−0.83	−0.40
EX6	0.76	2.72	1.53	0.31	0.86	1.03
EX7	2.45	20.40	12.45	4.20	−0.86	−0.54
EX8	71.77	296.55	147.03	51.68	0.57	−0.64
EX9	0.000006	0.000283	0.000068	0.000022	3.51	28.03
EX10	294.57	5154.40	1997.05	1476.69	0.87	−0.57
EX11	0.22	16.51	6.07	3.44	0.51	−0.26
EX12	0.0000	0.000388	0.000064	0.000031	2.84	24.56

Notes: Y: the number of hospital beds per ten thousand people; SE1–SE20: socioeconomic variables; EX1–EX12: environmental variables; Std. Deviation: standard deviation; Skewness: a measure of the direction and degree of skew in the distribution of statistical data. A normal distribution has a skewness value of zero; Kurtosis: a statistic that describes the steepness of the data distribution pattern. A normal distribution has a kurtosis value of zero.

**Table A2:** Multicollinearity evaluation for the socioeconomic (SE1–SE20) and environmental (EX1–EX12) variables.

Socioeconomic	VIF	Selection	Environment	VIF	Selection
SE1	6.67	N	EX1	1.68	Y
SE2	6.19	N	EX2	1.30	Y
SE3	7.68	N	EX3	3.00	Y
SE4	39.34	N	EX4	39.06	N
SE5	24.53	N	EX5	56.51	N
SE6	2.50	Y	EX6	2.95	Y
SE7	8.97	N	EX7	47.18	N
SE8	20.72	N	EX8	7.69	N
SE9	39.99	N	EX9	1.24	Y
SE10	1.55	Y	EX10	78.75	N
SE11	17.04	N	EX11	2.50	Y
SE12	11.16	N	EX12	1.20	Y
SE13	50.85	N			
SE14	18.11	N			
SE15	61.97	N			
SE16	25.15	N			
SE17	1.37	Y			
SE18	4.63	Y			
SE19	1.36	Y			
SE20	1.17	Y			

Notes: VIF: variance inflation factor; variables screening threshold: VIF < 5; Y denotes yes, and N denotes no.
